# Relevance and limits of patient surveys within humanitarian primary health care in Germany

**DOI:** 10.1093/eurpub/ckac129.718

**Published:** 2022-10-25

**Authors:** A Dieterich

**Affiliations:** 1 Catholic University of Applied Social Science, Berlin, Germany; 2 Alice-Salomon-University of Applied Sciences, Berlin, Germany

## Abstract

**Background:**

In Germany, distinct parts of the population, such as undocumented persons, uninsured German nationals, EU citizens without employment and asylum seekers, do not have adequate access to health care. Civil society organisations provide humanitarian medical aid for these individuals. However, the offered care is not comprehensive, but depends on volunteers and is restricted to acute conditions. The local initiatives are often underfinanced. To receive funding, they experience increasing pressure to provide evidence of the impact of their work.

**Methods:**

The applied research project was conducted in cooperation with a German medical Non-Government Organization. A routine patient survey, including items on patient satisfaction and patient reported outcomes, was developed and implemented. The NGO aimed to use it as an instrument for patient participation as well as to strengthen their impact assessment.

**Results:**

Survey results show high satisfaction levels and a reported increase in health status and system competence. They provide valuable answers for organisational development and fundraising but do not correlate with the extent of care provided.

**Conclusions:**

Patient surveys are particularly helpful in humanitarian care to legitimatize the efforts of compensatory initiatives. Since the affected persons still suffer from undersupply, results are not a valid measure of care standards but rather demonstrate appreciation for the mere existence of volunteer help. For an effective participation, one should not confound involvement in surveys with access to care. To illuminate existing barriers, alternative methodical approaches such as qualitative multilevel case studies are promising, as they allow data on local support settings to be linked with individual care histories and underlying institutional frameworks.

